# The Data Mounts: 261 Cleft Lifts for Complex Pilonidal Disease and Excisional Failures

**DOI:** 10.7759/cureus.50174

**Published:** 2023-12-08

**Authors:** Brian Shrager

**Affiliations:** 1 Surgery, Pilonidal Treatment Center of New Jersey, Hackettstown, USA

**Keywords:** pilonidal wound, bascom cleft-lift, pilonidal excision, pilonidal sinus, pilonidal cyst

## Abstract

Introduction: The Bascom cleft-lift procedure is a superior approach for treating pilonidal disease. The purpose of this study was to establish healing time after cleft-lift, operative success, and any associated clinical or operative variables.

Methods: The study group comprises all patients who underwent cleft-lift procedures at our center between December 2021 and February 2023. Many clinical and operative variables were collected before surgery. Postoperatively, patients were examined every two weeks until full epithelialization was achieved; thereafter, they were seen at 6, 16, and 30 months and as needed for recurrence surveillance. A successful cleft-lift was defined as one that fully healed by 120 days and showed no recurrence within 18 months of follow-up. Patients with failed cleft lifts were offered revision.

Results: In total, 261 cleft -lifts were performed in 258 patients. Of these patients, 40.3% had at least one previous excisional surgery and 19.4% had a chronically open surgical wound. The median follow-up time was 19.8 (6.5 to 25.5) months. There were a total of 12 failed cleft-lifts, yielding an operative success rate of 95.4%. Recurrence was detected in two (0.08%) cases. The median healing time was 43 (15-387) days and did not differ by any covariate. Previous Limberg flap surgery and a shorter distance from the inferior extent of the wound/disease to the anal mucosa were associated with decreased operative success.

Conclusion: Our data reinforce that the cleft-lift procedure is a highly successful cure for this disease and its surgical failures. Notably, the operation was a successful cure for many patients with extensive disease and previously failed excisional surgeries, including flap reconstructions.

## Introduction

Pilonidal disease is an acquired infectious disorder of the gluteal cleft skin. Pathogenesis is observed in traumatized dilated follicles along the midline crease. Through these so-called “pits”, thick hair shafts burrow deep into the epidermis. When anaerobic bacteria enter the fray, subcutaneous abscesses and/or lateral sinus tracts develop. Most commonly affecting males in the second and third decade of life, it brings pain and suppurative drainage symptoms to those affected, often with an emotional and social toll [1].

Since Mayo first described the disease in 1830, the cure has perplexed the most adept providers. For much of this time, excision of an underlying “cyst”, postulated by some to be congenital, has formed the cornerstone of definitive management, with the resulting midline wound either primarily closed or left open to heal by secondary intention. Unfortunately, these excisional methods are notorious for their unfavorable results, with high early wound failure and late recurrence rates reported [2-6].

Improvements in surgical outcomes began with the early work of Greek surgeon G.E. Karydakis in 1973. By attributing part of the pilonidal disease to the vulnerable midline crease skin, he devised the “Karydakis flap”, in which an off-midline elliptical incision aims to excise that skin, thereby lateralizing the surgical wound and positioning less vulnerable skin at the base of the gluteal cleft. The lateralized surgical wound sees higher ambient oxygen levels, lower bacterial load, and decreased shearing forces, all of which contribute to more reliable healing and lower recurrence rates. The operation has been repeatedly shown in both randomized controlled trials and meta-analyses to have very acceptable short-term and long-term results [7-12].

American surgeon John Bascom further tailored this off-midline approach [2]. He added the preoperative inscription of the gluteal skin contact lines, obtained in the standing position, which helped to guide both the extent of lateralization and subcutaneous flap dissection. Normal tissue was minimally excised, and any dead space was reduced [3]. Most importantly, as reflected in its name, the so-called “cleft-lift” clearly defines the shallowing of the gluteal cleft as its primary objective. In a recent meta-analysis using data from 80,000 patients over a 180-year period, cleft-lift was found to be the superior approach with regard to long-term recurrence rate [13]. Immerman SC [3], Bascom J [14] and Sternberg J [15] most eloquently describe this procedure and establish the desired results. Viewed through their respective lenses, the cleft-lift can be seen less as an excision of a cyst and its associated inflammatory tissue and more as an interruption of an anatomy-based disease mechanism [3,15]. The recurrence rate of cleft-lift does not exceed 5% in these recent high-volume series.

The primary purpose of this retrospective cohort study was to determine the healing time and cure rate following the cleft-lift procedure. Clinical and operative variables were investigated for any effect on the healing time and cure rate. The secondary purposes were to establish postoperative complication rates and long-term recurrence rates of the cleft-lift procedure.

## Materials and methods

The procedures in this study, including obtaining informed consent, were conducted in accordance with the ethical standards set forth in the Helsinki Declaration of 1975. All patients undergoing the cleft-lift procedure at our Center between December 2021 and February 2023 comprise the study group. The criteria for offering the cleft-lift procedure were (a) two or more abscesses requiring drainage, (b) one or more chronic sinus tract(s), or (c) nonhealing pilonidal excisional wound. During the entire study period, no prospective patients were excluded from consideration based on the extent of disease, perianal extent of wound/disease, previous excisional surgery, or comorbidity. Patients with acute abscesses were sometimes approached with a single-stage cleft lift, provided that all erythema could be incorporated into the excision. Other abscess patients were treated with index lancing followed by interval cleft-lift once the erythema had fully resolved.

**Figure 1:**
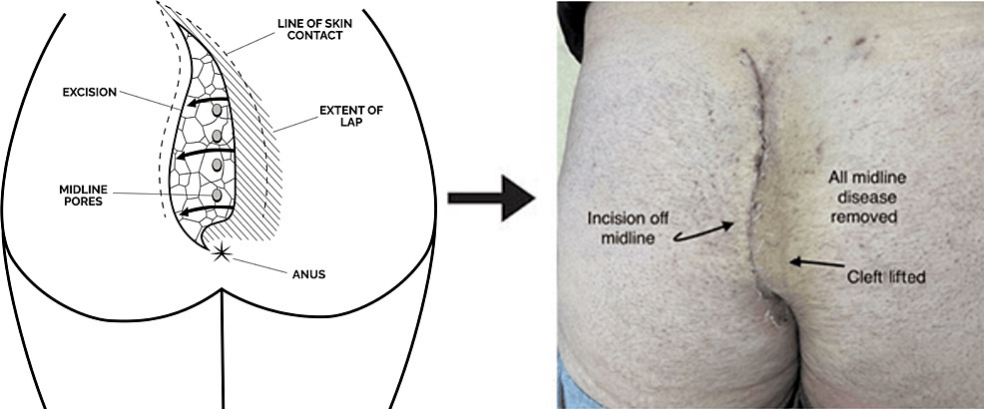
Cleft lift procedure Diagram shared with permission from pilonidal.com. The lines of gluteal skin contact are obtained either in the standing position or when prone by compressing the buttocks together and gently passing the skin marker along the line of skin contact. When released, a wishbone-shaped line results, indicative of the intact gluteal cleft.

**Figure 2:**
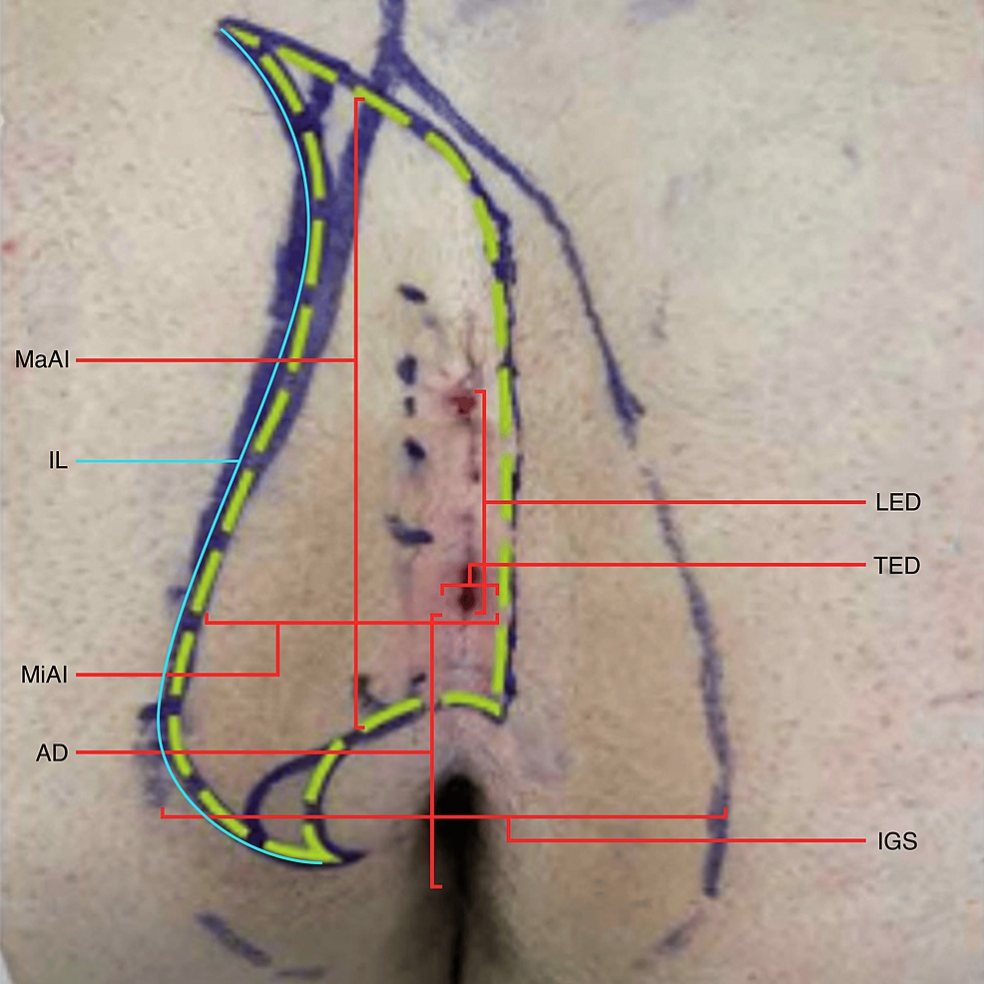
Measurements taken prior to incision The dashed green line represents the initial incision marking, which would be tailored after flap mobilization based on the assessment of tension. The broader wishbone-shaped marking represents the lines of gluteal skin contact obtained in the standing position. LED, longitudinal extent of disease; TED, transverse extent of disease;  IGS, intergluteal span; MaAI, major axis of the incision; MiAI, minor axis of incision; IL, incision length; AD, anus distance, i.e., distance from the inferior aspect of wound/disease to anal verge mucosa; IGS, intergluteal span.

We collected clinical, anatomical, disease-specific, and incision-specific variables in a database on the date of surgery (Table 1, Figures 1, 2). The variables were age, sex, height, weight, body mass index, cigarette smoking, previous and type of excisional surgery, disease duration, number of previous abscesses requiring drainage, presence of active erythema, presence of sinus tract or open surgical wound. A key anatomical variable measured and analyzed was the intergluteal span, which can be seen as a quantitative expression of cleft depth; it is the measured distance from one gluteal contact line to the contralateral contact line at the cephalad aspect of the anal verge. A key disease-specific variable measured and analyzed was the distance from the inferior aspect of the wound/disease to the anal canal mucosa.

**Table 1:** Descriptive data

Cohort (n=261)	n	%
Gender		
Male	199	77.1
Female	59	22.9
Smoking		
Yes	20	7.8
No	238	92.3
Any previous excision	N	%
Yes	104	40.3
No	154	59.7
Most recent excisional surgery		
midline closure	39	37.5
lay-open	33	31.7
pit-picking	11	10.6
cleft-lift	7	6.7
Limberg flap	4	3.8
Gips	2	1.9
Karydakis flap	1	1.0
closure attempt	1	1.0
Endoscopic pilonidal sinus treatment (EPSiT)	1	1.0
secondary closure	1	1.0
sinusectomy	1	1.0
skin grafting	1	1.0
transverse closure	1	1.0
z-plasty	1	1.0
Open wound		
Yes	50	19.4
No	208	80.6
Number of abscesses		
0	131	51.4
1	60	23.5
2	36	14.1
3	12	4.7
4	6	2.4
5	2	0.8
>5	8	3.1
Sinus tract		
Yes	124	48.1
No	134	51.9
Erythema		
Yes	16	6.2
No	242	93.8

**Figure 3:**
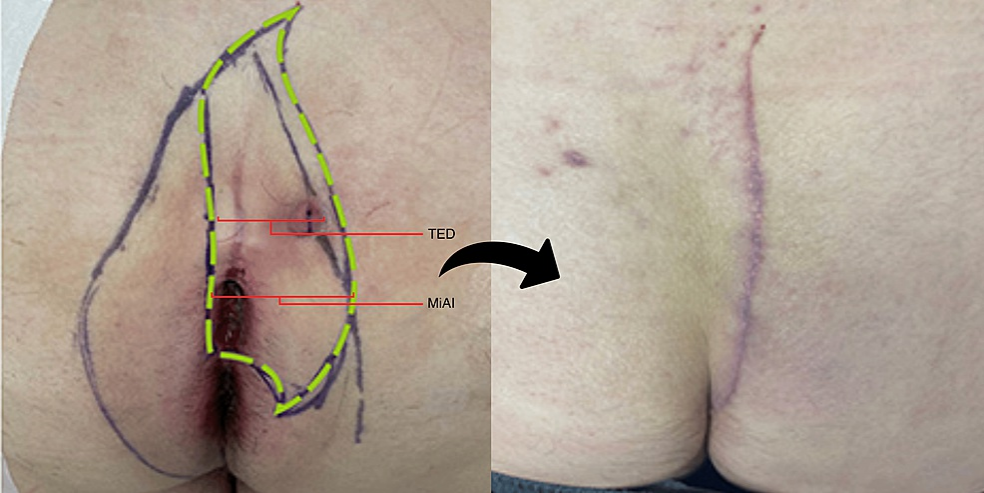
Wide index cleft-lift The left pane shows preoperative marking. The broader wishbone-shaped marking represents the line of gluteal skin contact, which informs the initial incision marking, indicated by the green dashed line.  The fairly long transverse extent of the disease (TED) necessitated a fairly wide excision so that the entire sinus tract from the midline pits to the pyogenic granuloma could be clearly incorporated into the excision. The right pane shows cleft-lift at six weeks of healing.

**Figure 4:**
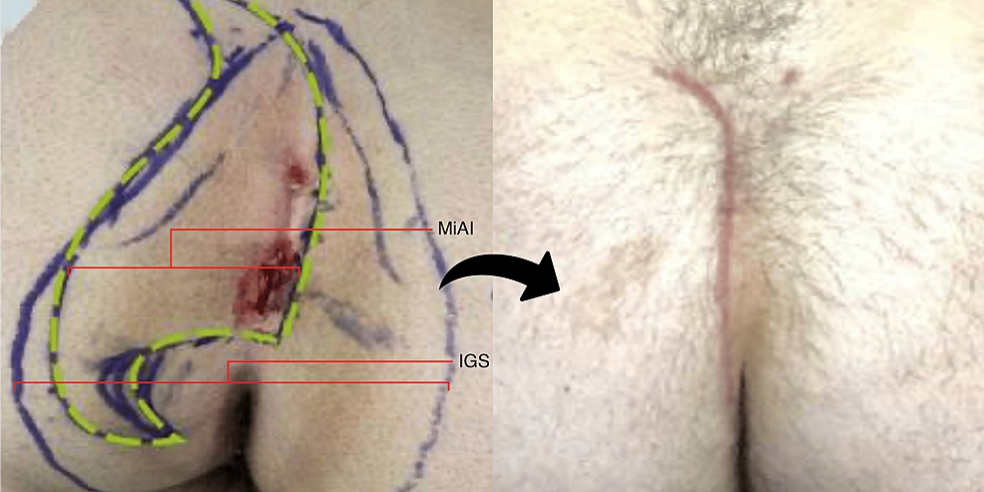
Index cleft-lift in the deep cleft The left pane shows preoperative marking. The broader wishbone-shaped marking represents the line of gluteal skin contact. The patient had a naturally deep cleft as indicated by the long intergluteal span (IGS), necessitating a wide excision, indicated by the green dashed line with its long minor axis of incision (MiAI). If the anatomy is not respected in this way, the surgery is doomed to failure. The right pane shows the fully healed recurrence-free cleft-lift at 16 weeks after surgery.

The cleft-lift was performed as previously described (Figures 3, 4) [3,14,15]. Specific to this technique, all pathological tissues, including the abscess cavity wall, sinus tract, sinus-associated pyogenic granuloma, and non-healing granulation tissue, were routinely excised in the operation (Figure 3). Like Immerman SC [3], following flap mobilization and closure of the deepest layer, the flap was moved into its planned position, and the incision line was tailored based on an assessment of tension, yielding an additional skin margin which was measured and entered into our analysis. Following the excision of a chronic open wound, the wound base was bluntly debrided using the coarse aspect of an iodine-soaked scrub sponge. A closed-suction drain was placed and remained in situ until the drainage was less than 20 cc/day for two consecutive days. Each specimen was sectioned and cultured ex vivo to treat potential infectious complications. Postoperative oral antibiotics were not routinely used except in the acutely infected cases mentioned above. Postoperatively, patients were followed in our clinic every two to three weeks until full epithelialization was achieved without granulation tissue or scab. Patients were monitored for wound complications and treated accordingly. Seroma was defined as transudative fluid collection requiring either surgical or percutaneous drainage or a drain duration longer than 14 days. Abscess was defined as the exudative fluid collection and wound infection was defined as cellulitis without fluid collection. Wound dehiscence was defined as a separation of at least 5 cm of the incision requiring re-approximation either in the office or the operative suite. Many patients had minor caudal separations that were conservatively managed until full healing was achieved; these were not reported as complications. Once full healing was achieved, patients were followed up at 6, 16, and 30 months and as needed as a matter of recurrence surveillance.

The median healing time was calculated using Kaplan-Meier estimates, and log-rank tests were performed to examine differences by covariates. A successful cleft-lift was defined as one that fully healed by the four-month (120 days) mark and showed no recurrence within 18 months of follow-up. Odds ratios and 95% confidence intervals were calculated, and Chi-square tests were used to examine the relationship between covariates and operative success. Patients with failed cleft lifts were offered revision.

## Results

Over a 14-month period, 261 cleft lifts were performed on 258 patients. The median follow-up time was 19.8 (6.5 to 25.5) months. The majority of patients were male (77.1%) and 104 (40.3%) had at least one previous excisional surgery (Table 1), most commonly midline closure (37.5% of previous surgeries) and the lay-open approach (31.7%). Fifty (19.4%) patients had chronically open surgical wounds, some extending very close to the anal mucosa. Approximately half (48.5%) had a history of multiple abscesses, 124 (48.1%) had one or more sinus tracts, and 16 (6.2%) had an acute pilonidal infection (erythema and/or purulence) at the time of surgery. The mean age at surgery was 27.5 years, and the median disease duration was 29 (51-900) months. There were 62 patients (23.7%) with short-term complications (Table 2), the most common being wound infection (4.7%) and wound dehiscence (5.4%). Abscess was postoperatively observed in five (1.9%) patients. The median healing time was 43 (15-387) days and did not differ by any covariate (Table 3).

**Table 2:** Postoperative complications

Postoperative Complications	n	%
Wound Infection	12	4.7
Wound dehiscence	12	5.4
Suture granuloma	11	4.3
Abscess	5	1.9
Scar ulceration	9	3.5
Seroma	2	0.8
Hematoma	2	0.8
Constipation	2	0.8
Hemorrhoids	2	0.8
Retained drain	2	0.8
Anocutaneous fistula	1	0.4
Chronic pain	1	0.4
Interoperative pulmonary edema	1	0.4

**Table 3:** Predictors of healing time

	Healing time (days)	
Gender	Median	p-value
Male	41	0.17
Female	47	
Smoking		
Yes	48	0.82
No	42	
Any previous excision		
Yes	46	0.52
No	41	
Previous Limberg flap surgery (most recent excision)		
Yes	41	0.19
No previous surgery	51.5	
Previous midline closure surgery (most recent excision)		
Yes	43	0.50
No previous surgery	41	
Open surgical wound		
Yes	41	0.23
No	48	
Number of abscesses		
0	45	0.14
1	31	
2	41	
3	31.5	
4	39.5	
5	45	
>5	55	
Sinus tract		
Yes	41.5	0.38
No	43	
Erythema		
Yes	43	0.34
No	43	
Age at surgery		
<25	43	0.55
25-40	43	
>=40	28	
Body Mass Index (kilogram/meter^2^)		
<25	43	0.17
25-<30	42	
≥30	39.5	
Intergluteal span (centimeters)		
≥11.7	43	0.39
<11.7	41.5	
Distance from the anal verge (centimeters)		
≤3	48	0.11
3-6	43	
>6	37	
Longitudinal extent of disease (centimeters)		
≥4.8	43	0.87
<4.8	43	
Transverse extent of disease (centimeters)		
≤1	43	0.35
1-2	44	
>2	43	
Major axis of incision (centimeters)		
≥15.2	43	0.72
<15.2	41.5	
Minor axis of incision (centimeters)		
≥5.3	43	0.67
<5.3	41	
Incision length (centimeters)		
≥17.6	43	0.78
<17.6	41	
Skin Margin (centimeters)		
≥1.7	43	0.79
<1.7	42	
Operative time (minutes)		
≥95	43	0.96
<95	41	
Disease duration (months)		
≤12	41	0.76
12-36	41	
≥36	43	

Two (0.08%) patients had a recurrence within 18 months, and eight patients did not heal within four months and were considered non-healers. One patient with a recurrence was successfully revised. Two of the non-healers underwent revision, and each subsequently failed a second time by nonhealing, for a total of 12(4.6%) failed cleft-lifts. Our analysis showed that operative success did not differ by sex, age, body mass index, smoking, presence of open wound, sinus tract, acute erythema, extent of disease burden at the skin level, major and minor axes of incision marking, incision length, additional skin margin taken during the tailoring portion of the operation, or duration of disease (Table 4).

**Table 4:** Predictors of operative success

	N	%	Odds Ratio	95% Confidence Interval	p
Gender					
Male	193	97.0	1.13	0.22-5.75	0.88
Female	57	96.6			
Smoking					
Yes	20	100.0	1.51	0.08-27.15	0.41
No	230	96.6			
Any previous excisional surgery					
Yes	100	96.2	0.67	0.16-2.73	0.57
No	150	97.4			
Previous Limberg flap surgery (most recent excision)					
Yes	3	75.0	0.08	0.01-0.95	0.01
No previous surgery	150	97.4			
Previous midline closure surgery (most recent excision)					
Yes	38	97.4	1.01	0.11-9.33	0.99
No previous surgery	150	97.4			
Open wound					
Yes	47	94.0	0.39	0.09-1.67	0.19
No	203	97.6			
Number of abscesses					
0	126	96.2			0.83
1	60	100.0			
2	34	94.4			
3	11	91.7			
4	6	100.0			
5	2	100.0			
>5	6	100.0			
Sinus tract(s)					
Yes	120	96.8	0.92	0.23-3.77	0.91
No	130	97.0			
Erythema					
Yes	16	100.0	1.20	0.07-21.64	0.46
No	234	96.7			
Intergluteal span (centimeters)					
≥ 11.7	125	96.9	1.03	0.25-4.22	0.96
<11.7	121	96.8			
Longitudinal extent of disease (centimeters)					
≥ 4.8	119	95.2	0.33	0.06-1.66	0.16
<4.8	121	98.4			
Major axis of incision (centimeters)					
≥ 15.2	126	96.2	0.63	0.15-2.69	0.53
<15.2	120	97.6			
Minor axis of incision (centimeters)					
≥5.3	132	96.4	0.67	0.16-2.87	0.59
<5.3	118	97.5			
Incision length (centimeters)					
≥17.6	120	95.2	0.33	0.06-1.64	0.15
<17.6	123	98.4			
Additional skin margin (centimeters)					
≥1.7	128	97.7	1.89	0.44-8.08	0.39
<1.7	113	95.8			
Operative time (minutes)					
≥95	119	96.8	0.94	0.23-3.86	0.94
<95	126	96.9			
Age at surgery (years)					0.09
<25	114	94.2			
25-40	107	100.0			
>=40	29	96.7			
Body Mass Index (kilogram/meter^2^)					
<25	69	98.6			0.25
25-<30	99	97.1			
≥30	82	95.4			
Distance from anal verge (centimeters)					
≤3	86	93.5			0.02
3-6	106	98.2			
>6	48	100.0			
Transverse Extent of Disease (centimeters)					
≤1	99	98.0			0.78
1-2	63	94.0			
>2	78	97.5			
Disease Duration (months)					
≤12	67	94.4			0.17
12-36	76	97.4			
>36	105	98.1			

Previous excisional surgery was not associated with operative success (odds ratio (OR) 0.67, 95% CI 0.16-2.73, p=0.57), nor were those with previous midline closure surgery less likely to have a successful surgery (OR 1.01, 95% CI 0.11-9.33, p=0.99). Previous Limberg flap surgery was associated with a reduced likelihood of success (OR 0.08, 95% 0.01-0.95, p=0.01), although the number was small (n=4). The other significant predictors of operative success were the distance of the inferior aspect of the wound or disease from the anal canal mucosa; those with a distance of ≤3 cm were less likely to have a successful surgery (93.5%) than those with a distance of at least 6 cm of intact skin in this area (100% successful, p=0.02).

## Discussion

**Figure 5:**
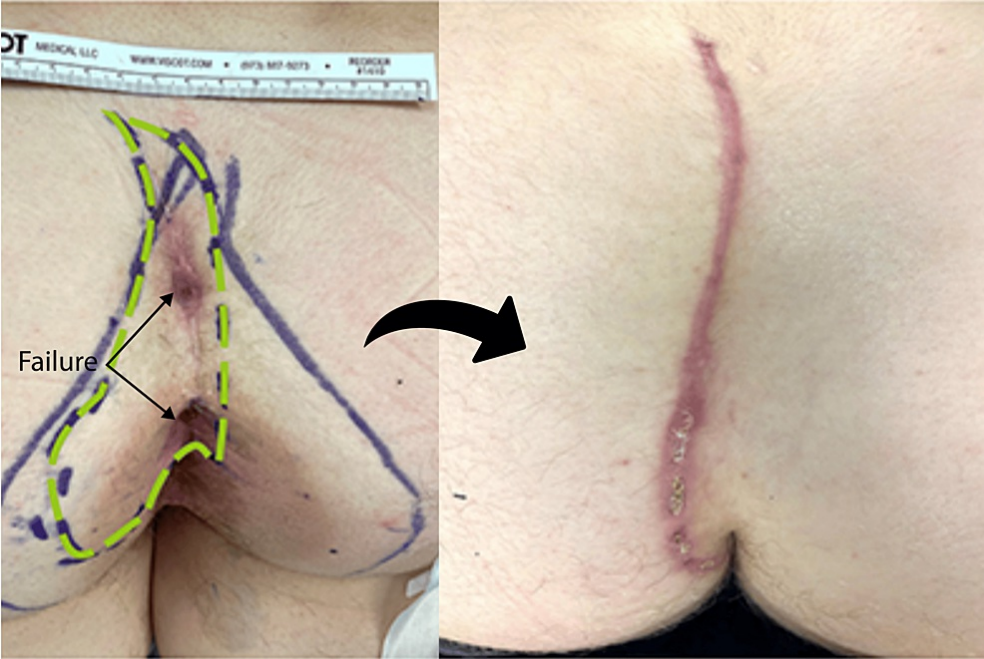
Revision of a failed cleft-lift The patient was told that the previous excision was a ”mini-cleft-lift”. Because only a short segment of the cleft was lifted, the anatomy was actually worsened, and the patient failed above and below this “lifted” segment. The left pane shows preoperative marking with the green dashed line indicating the initial incision marking. The broader wishbone-shaped marking connotes the line of gluteal skin contact, indicating a largely intact and non-lifted cleft. The green dashed line is the initial incision marking, which would be later tailored after flap mobilization.  The right pane shows the fully lifted cleft almost fully healed at six weeks.

Our data reinforces the established concept that the cleft-lift procedure is a highly successful cure for pilonidal disease. Our overall success rate of 95.4%, including a low recurrence rate of 0.08%, is in line with recent high-volume series [3,15]. Our analyses revealed that operative success was independent of a multitude of clinical, disease-specific, and incision-specific variables. Notably, the operation was a successful cure for many patients with previously failed excisional surgeries, including poorly performed cleft lifts (Figure 5).

**Figure 6:**
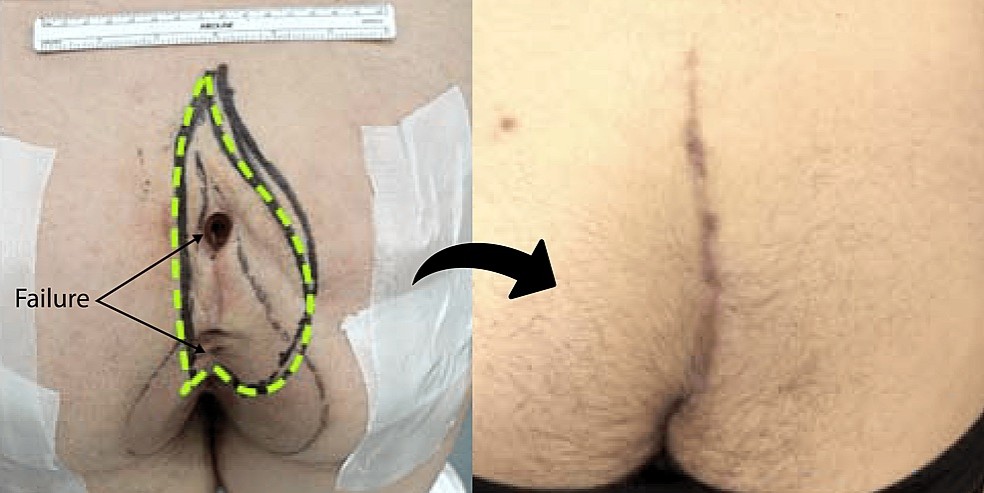
Revision of a failed Limberg flap The left pane shows the preoperative appearance of the failed rhomboid transposition flap. These reconstructions typically failed where the incision approached the midline. The dashed green line indicates the initial incision marking, which was planned to excise the entire flap. Also, the cleft-lift flap is always raised in native (not transposed) tissue. The broader wishbone-shaped marking represents the line of gluteal skin contact.  The right pane shows the fully healed clef lift at 18 weeks.

**Figure 7:**
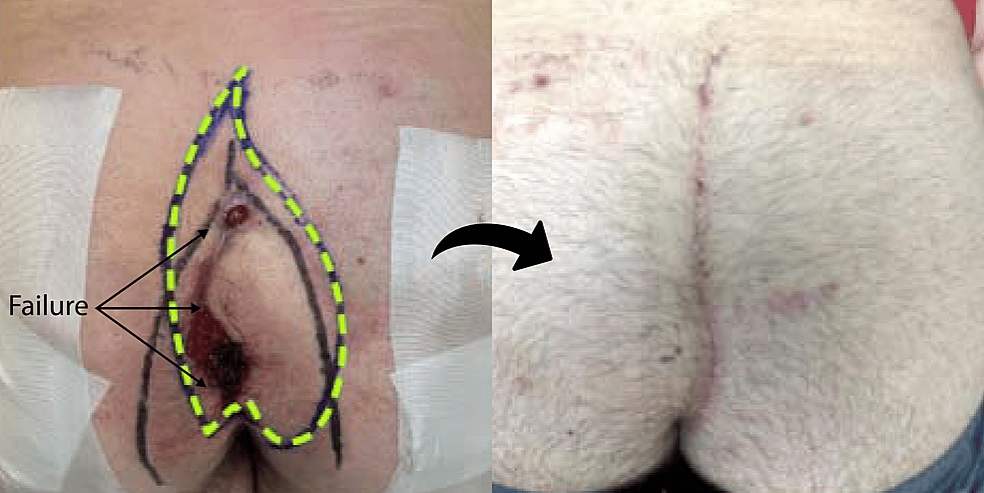
Revision of a second failed Limberg flap The right pane shows complete healing at 11 weeks.

One exception was failed Limberg flap reconstruction, which was associated with lower operative success. The reasons for this are not entirely clear. One might surmise that the wound edges are at risk for less hearty blood supply following the large fasciocutaneous rhomboid transposition flap of the Limberg reconstruction, but we mitigated this risk in our operative approach, which included full excision of the rhombus and raising of our subcutaneous flap only in native undisturbed tissues (see Figures 6, 7). With this surgical approach, we successfully cured many patients with failed Limberg flaps.

Interestingly, operative success and healing time were not associated with the extent of disease at the skin level or the area of skin excised, as indicated by the major and minor axes of incision, incision length, and additional skin margin taken during the tailoring phase of the operation. Figures 3, 4 typify these relatively aggressive skin excisions, the former dictated by the extent of disease at the skin surface, the latter dictated by the native anatomy, i.e., the deeper than average cleft. These findings seem to be inconsistent with the commonly held notion that wound tension underlies failure in pilonidal excision; it is the opinion of this author that mild tension is always present in a properly performed and durable cleft-lift.

**Figure 8:**
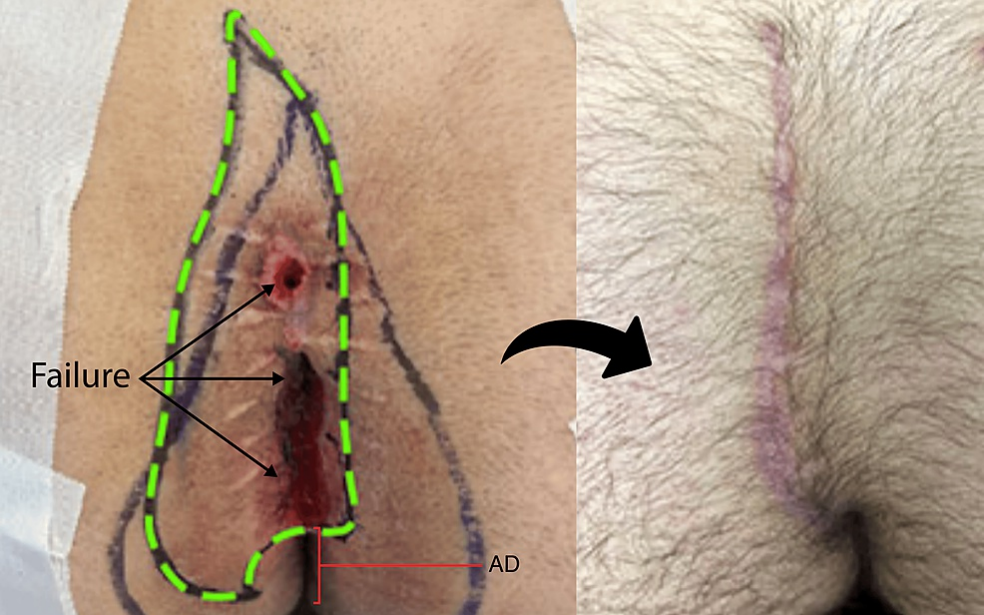
Low revisional cleft-lift The patient had a history of midline excision with the wound laid open, but never healed despite repeat attempts at closure. The left pane shows preoperative marking with the open wound extending to within 3 cm of the anal verge mucosa and a short anus distance (AD). The broader wishbone-shaped marking represents the line of gluteal skin contact obtained in the standing position. The green dashed line is the initial incision marking, which would be later tailored after flap mobilization. The large suture marks were a common finding in this clinical scenario, indicative of a previous surgeon attempting to deal with anticipated tension. The right pane shows the fully healed cleft lift at approximately 18 weeks; the caudal aspect of the incision is seen approaching the anus from a 10 to 11 o’clock orientation as is preferred for healing purposes.

One positive predictor of operative success was the distance between the bottom of the wound and the anal canal mucosa. This predictor was echoed in Immerman’s larger cohort [3]. Our failures in these low revisional or index cases were generally seen in the form of surgical wounds that did not fully heal by the four-month mark; these wounds were almost exclusively problematic in their perianal aspect, reminding us of the importance of vigilant postoperative care in these situations. Patients with slow-healing wounds at the perianal aspect were treated with a specially compounded metronidazole-based cream topically applied twice daily and aeration of this aspect of the wound with a twice-folded gauze wedge placed just below the slowly healing aspect of the wound. Bidet for post-defecation hygiene was encouraged. Activity, bathing, or sitting restrictions were not imposed on the patients during this last phase of healing, making this part of the recovery process more tolerable. Assuming that the incisions in these cases approached the anal verge from a 10 to 11 o’clock position (or a 2 to 3 o’clock position for right-sided cleft-lift), these wounds almost always healed with more time. With these surgical and postoperative strategies, we were still able to successfully revise 93.5% of our patients with “edge-of-anus” excisional wounds (Figure 8).

The median healing time for cases with edge-of-anus involvement was 48 days compared to cases with at least 6 cm of intact skin between the inferior extent of disease/wound and anal verge (37 days), but this did not achieve statistical significance (p=0.11). Our surgical successes notwithstanding, this finding reminds us of the morbidity of non-cleft lift excisional surgeries, which tend to leave progressively lower wounds in failure [3].

Finally, there were low rates of postoperative infectious complications, specifically abscesses (1.9%) and wound infections (4.7%). These were observed in the absence of postoperative antibiotics used in an empiric way. Surprisingly, none of the acutely infected one-stage cleft-lifts developed either of these complications. This latter finding seems to contradict the commonly held notion that acute pilonidal abscess should be surgically addressed in a staged manner, that is, drainage followed by an interval excisional procedure. The author strongly believes in the prompt tailoring of postoperative oral antibiotic therapy to operative culture results, perhaps contributing to our success in these acute cases. More broadly, these findings call into question the utility of routine postoperative oral antibiotic therapy, which is not without side effects.

The study was limited by a shorter than desired follow-up time. Our median follow-up time of 19.8 months was certainly sound and is amongst the highest reported in the literature [7-12]. From a logistical standpoint, long-term patient follow-up after a specialized and high-quality cleft lift was found to be challenging. Many patients had travelled more than several hundred miles to our Center for their surgery and, having usually been completely cured of this benign skin disease and now asymptomatic for several years, repeat travel demands were often prohibitive for many. To be more confident in our reported recurrence rate of 0.08%, it seems reasonable to this author to achieve a longer median follow-up. It seems necessary that future studies expand the use of telemedicine supplemented with high-resolution digital images to achieve this level of recurrence surveillance.

## Conclusions

The Bascom cleft-lift is once again shown to be the superior curative approach for all forms of gluteal cleft pilonidal disease including excisional failure and unsuccessful flap reconstructions. Disease presentations of all forms, including those with broad skin involvement and perianal extension, can be reliably addressed with the operation. Patients should not be hurried into the operation but offered it only if strict criteria are met, because duration of disease is a variable found to be of no influence on healing time or operative success rate. Although difficult with a benign skin disease that has likely been cured, future studies should attempt to establish the greatest length of follow-up time to confirm low recurrence risk.
